# Activation of BDNF–TrkB Signaling in Specific Structures of the Sheep Brain by Kynurenic Acid

**DOI:** 10.3390/cells13231928

**Published:** 2024-11-21

**Authors:** Katarzyna Roszkowicz-Ostrowska, Patrycja Młotkowska, Elżbieta Marciniak, Michał Szlis, Marcin Barszcz, Tomasz Misztal

**Affiliations:** The Kielanowski Institute of Animal Physiology and Nutrition, Polish Academy of Sciences, Instytucka 3 Str., 05-110 Jabłonna, Poland; k.roszkowicz@ifzz.pl (K.R.-O.); p.mlotkowska@ifzz.pl (P.M.); e.marciniak@ifzz.pl (E.M.); m.szlis@ifzz.pl (M.S.); m.barszcz@ifzz.pl (M.B.)

**Keywords:** kynurenic acid, intracerebroventricular infusion, BDNF, TrkB, sheep brain

## Abstract

Fluctuations in kynurenic acid (KYNA) and brain-derived neurotrophic factor (BDNF) levels in the brain reflect its neurological status. The aim of the study was to investigate the effect of transiently elevated KYNA concentrations in the cerebroventricular circulation on the expression of BDNF and its high-affinity tropomyosin-related kinase receptor B (TrkB) in specific structures of the sheep brain. Intracerebroventricularly cannulated anestrous sheep were subjected to a series of four 30 min infusions of KYNA: 4 × 5 μg/60 μL/30 min (KYNA20, *n* = 6) and 4 × 25 μg/60 μL/30 min (KYNA100, *n* = 6) or a control infusion (*n* = 6), at 30 min intervals. Sections of the hippocampal CA3 field, amygdala (AMG), prefrontal cortex (PCx), and the hypothalamic medial-basal (MBH) and preoptic (POA) areas were dissected from the brain immediately after the experiment. The highest concentration of BDNF protein was found in the CA3 field (*p* < 0.001), which was 8-fold higher than in the AMG and 12-fold higher than that in the PCx (MBH and POA were not analyzed). The most pronounced BDNF mRNA expression was observed in the MBH, followed by the PCx, POA, AMG and CA3, while the highest abundance of TrkB mRNA was recorded in the AMG, followed by the MBH, PCx, CA3, and POA. KYNA increased (*p* < 0.05–*p* < 0.01) BDNF protein levels and the expression of its gene in the brain structures were examined, with the effect varying by dose and brain region. KYNA, particularly at the KYNA100 dose, also increased (*p* < 0.01) *TrkB* gene expression, except for the AMG, where the lower KYNA20 dose was more effective (*p* < 0.01). These findings suggest a positive relationship between KYNA levels in the cerebroventricular circulation and BDNF–TrkB expression in specific brain regions in a sheep model. This indicates that a transient increase in the CSF KYNA concentration can potentially restore BDNF production, for which deficiency underlies numerous neurological disorders.

## 1. Introduction

Brain-derived neurotrophic factor (BDNF) is the dominant member of the neurotrophin protein family, which also includes nerve growth factor, neurotrophin-3 and neurotrophin-4 [1]. Neurotrophins regulate neuronal survival and neuroplasticity, playing important roles in the growth, differentiation, and repair of neurons [1,2]. BDNF has also been shown to modulate neuronal transmission in brain structures critical for learning and memory processes, while exerting neuroprotective effect in adverse conditions, such as glutamatergic stimulation, cerebral ischemia, hypoglycemia, or neurotoxicity [3,4]. Along with its signaling partners and other trophic factors, such as glial-derived neurotrophic factor and vascular endothelial growth factor, BDNF influences neurogenesis and numerous accompanying processes, like gliogenesis and angiogenesis [2,5]. In mammals, the capacity for neurogenesis in the brain persists throughout postnatal and adult life, primarily in two neurogenic niches located in the subgranular zone (SGZ) of the hippocampal dentate gyrus (DG) and the subventricular zone (SVZ) of the lateral ventricles [6]. Lower innate levels of neurogenesis have also been found in other brain regions, including the basal forebrain, striatum, amygdala, substantia nigra, and hypothalamus [7,8]. Adult neurogenesis is believed to play an important role in processes, such as learning and memory, emotions, stress, depression, and response to injury [9]. This process can be influenced by environmental factors and experiences, indicating that newly generated neurons can mediate interactions with the environment [10,11]. The disruption or inhibition of adult neurogenesis has been linked to changes that may impair neuronal functions and, consequently, the development of dementia or depression [12].

BDNF is abundantly expressed in the immature and adult mammalian brain, and its messenger RNA (mRNA) and protein levels in various structures increase dramatically during the postnatal period [13]. Neurotrophin is initially synthesized as a precursor protein (pro-BDNF), which is then cleaved into its mature form and stored in secretory vesicles. The co-localization of the cleaved pro-peptide region and mature BDNF in secretory vesicles of hippocampal neurons indicates that this conversion occurs directly within neurons [14]. Mature BDNF is released locally from both axonal and dendritic compartments in a process dependent on neuronal activity [15]. The neurotrophic effects of BDNF are primarily mediated by its high-affinity tropomyosin-related kinase receptor B (TrkB), while pro-BDNF binds preferentially to p75NTR. The activation of BDNF–TrkB signaling stimulates different intracellular pathways that regulate the expression of genes encoding proteins responsible for the plasticity of neurons, resistance to stress, and cell survival, among others [3,16].

Studies using different animal models and humans show that abnormalities related to BDNF synthesis and secretion, causing a significant decrease in its levels in the brain, are associated with many neurological disorders and central nervous system (CNS) diseases [12,17]. Decreased levels of BDNF protein and mRNA expression have been observed in the brains of individuals suffering from Alzheimer’s and Huntington’s diseases, as well as depression and schizophrenia [17,18,19,20]. These abnormalities may not be directly related to the expression of BDNF but may also result from age-related alterations in the production of endogenous substances that modulate the activity of the excitatory glutamatergic system. Among such substances is kynurenic acid (KYNA), a neuroactive tryptophan (TRP) metabolite, for which fluctuating levels in the CNS may be related to neurotrophic activity and the course of adult neurogenesis. KYNA is the only known endogenous non-selective antagonist of all ionotropic receptors for excitatory amino acids in the mammalian brain, sensitive to N-methyl-D-aspartate (NMDA), kainic acid, and α-amino-3-hydroxy-5-methyl-4-isoxazolepropionic acid (AMPA) receptors [21]. These receptors play an indisputable role in neuronal excitability, firing patterns, plasticity, and behavior. While KYNA brain levels are elevated during the perinatal period in mammals, extracellular concentrations in adults generally remain in the low nanomolar range [22]. However, the reported elevations in KYNA concentrations in the adult brain have been associated with a broad spectrum of neurological and psychiatric disorders, particularly those linked to excitotoxicity [21]. Consequently, experimentally induced increases in KYNA levels in the brain have been shown to reduce toxic excitatory neurotransmission and inhibit some neurodegenerative changes [23]. On the other hand, high levels of KYNA were demonstrated to impair cognitive functions, as observed in schizophrenia, while reducing KYNA concentrations enhanced cognitive ability in rodents [24]. The mechanisms underlying these beneficial and detrimental effects are complex and may involve various types of receptors (not only for excitatory amino acids), signaling pathways, and neuroactive proteins, including BDNF [25].

The relationship between KYNA and BDNF in specific CNS pathologies remains unclear, and their interaction under physiological conditions is also poorly understood. Given this, the present study aimed to investigate the effect of transiently increased KYNA concentrations in the cerebroventricular circulation on the expression of BDNF and its receptor TrkB in specific areas of the sheep brain. The examined neuronal structures included the hippocampal CA3 field, amygdala (AMG), and prefrontal cortex (PCx), as well as the hypothalamic medial-basal (MBH) and preoptic (POA) areas. The sheep model was selected for the study, due to its higher degree of anatomical and structural homology to the human brain compared to commonly used smaller laboratory animals.

## 2. Materials and Methods

### 2.1. Animal Management

Eighteen Polish Longwool sheep (a breed showing reproductive seasonality), aged 1 year and weighing 55 ± 2 kg, were used in the experiment. The animals were bred at the Sheep Breeding Center of the Kielanowski Institute of Animal Physiology and Nutrition, Polish Academy of Sciences (Jablonna near Warsaw, Poland) under natural lighting conditions (52 °N, 21 °E). Sheep were fed twice daily according to their physiological status, with a diet based on pellet concentrate and hay, following the recommendations of the National Research Institute of Animal Production in Krakow-Balice (Poland) and the National Institute for Agricultural Research (France) [26]. During the experimental period, sheep were housed in individual pens with visual, olfactory, and tactile contact and were provided free access to water and mineral licks.

### 2.2. Third Ventricle (IIIv) Cannulation

One month before the experiment, the sheep underwent the surgical implantation of a stainless-steel guide cannula into the third ventricle (IIIv) of the brain (outer diameter: 1.2 mm, frontal position: 31.0 mm), as described previously [27]. Specifically, the implantation was performed under general anesthesia (xylazine: 40 mg/kg body weight, intravenously; xylapan and ketamine: 10-20 mg/kg body weight, intravenously; Bioketan, Vetoquinol Biowet, Pulawy, Poland), in accordance with the stereotaxic coordinate system for the sheep hypothalamus [28] and the procedure described by Traczyk and Przekop [29]. The guide cannula was fixed to the skull with stainless-steel screws and dental cement, and the external orifice of the canal was sealed with a stylet. After surgery, the sheep were injected for 4 days with antibiotics (1 g streptomycin and 1,200,000 IU benzylpenicillin; Polfa, Warszawa, Poland) and analgesics (metamizole sodium: 50 mg/animal; Biovetalgin, Biowet Drwalew, Drwalew, Poland, or meloxicam: 1.5 mg/animal; Metacam, Boehringer Ingelheim, Ingelheim am Rhein, Germany). The correct positioning of the cannula in the ventricle was confirmed in all sheep through cerebrospinal fluid (CSF) efflux surgery and a brain examination after slaughter.

### 2.3. Experimental Design and Tissue Collection

The experiment was performed in March during the natural anestrous season for this breed of sheep. The animals were randomly divided into three groups (*n* = 6 each) and infused into the IIIv with Ringer–Locke solution (RLs, control) or with one of two doses of KYNA (Sigma Chemical Co., St Louis, MO, USA) dissolved in RLs [27]. The treatment consisted of a series of four 30 min infusions, at 30 min intervals, from 10:00 to 14:00. The selected KYNA doses were as follows: lower—4 × 5 μg/60 μL/30 min (KYNA20), and higher—4 × 25 μg/60 μL/30 min (KYNA100), which were based on the scientific literature [22,25]. All infusions were delivered using a BAS Bee microinjection pump (Bioanalytical Systems Inc., West Lafayette, IN, USA) and calibrated 1.0 mL gas-tight syringes. During the treatments, sheep were kept in pairs in the experimental room in comfortable cages, where they could lie down and to which they had been acclimatized for three days prior to the experiment. Immediately after the experiment, sheep were slaughtered after prior pharmacological stunning (xylazine 0.2 mg/kg body weight and ketamine: 3 mg/kg body weight, intravenously), and the brains were rapidly removed from the skull. Following the separation of the median eminence, each brain was sagittally sectioned into the cerebral hemispheres. The isolated blocks of the hypothalamus (cut to a depth of 2 mm) were then dissected into two regions: POA and MBH, according to the stereotaxic atlas of the ovine hypothalamus [28]. The hippocampal CA3 field and AMG were dissected from the medial temporal lobe of the right hemisphere, as described previously [30]. Subsequently, approximately 2–3 mm-long sections, representing the PCx, were incised from the anterior frontal lobe of the cerebral cortex. All tissue incisions were performed on sterile glass plates placed on ice, and the collected structures were immediately frozen in liquid nitrogen and stored at −80 °C.

### 2.4. Tissue BDNF Concentration Assay

Frozen sections (CA3, AMG and PCx) were mixed with radioimmunoprecipitation assay (RIPA) buffer (0.5 M Tris-HCl, pH 7.4, 1.5 M NaCl, 2.5% deoxycholic acid, 10% NP-40, 10 mM EDTA) (Merck, Darmstadt, Germany) at a ratio of 1:10 (tissue to reagents), with aprotinin as protease inhibitor (10 IU/mL, Sigma-Aldrich, Saint Louis, MO, USA). Each tissue sample was homogenized using a laboratory homogenizer and ceramic beads. After 30 min of incubation on ice, the homogenates were centrifuged at 12,000× *g* for 10 min at 4 °C. The supernatants were then transferred to a new 1.5 mL Eppendorf tube and immediately stored at −80 °C for later use. The BDNF concentration in the homogenates was determined using the Biosensis Mature BDNF Rapid ELISA kit (BEK-2211, Biosensis Pty Ltd., Thebarton, Australia) according to the manufacturer’s protocol. Although originally designed for humans, mice, and rat studies, this ELISA kit is also suitable for quantifying mature BDNF in biological material obtained from other mammalian species, including sheep [31]. The assay demonstrated reproducibility with intra- and interassay CVs of 1% and 5%, respectively, with a minimum detectable dose of BDNF of less than 2 pg/mL. In addition, the total protein concentration in the tissue homogenates was analyzed spectrophotometrically using the Bradford method and the Bio-Rad Protein Assay Kit II (Bio-Rad, Hercules, CA, USA), according to the manufacturer’s instructions. The BDNF concentration in each homogenate sample was expressed as pg per mg of total protein.

### 2.5. Relative mRNA Abundance

Total RNA from hypothalamic and hippocampal tissues was isolated using the NucleoSpin RNA II kit (Macherey-Nagel, Düren, Germany), according to the manufacturer’s protocol. The concentration and purity of isolated RNA were quantified using a NanoDrop ND-1000 spectrophotometer (Thermo Fisher Scientific, Waltham, MA, USA). RNA integrity was electrophoretically verified on a 1.5% agarose gel stained with ethidium bromide. The TranScriba Kit (A&A Biotechnology, Gdynia, Poland) was used to synthesize cDNA as per the manufacturer’s instructions with 1 µg of total RNA in a reaction volume of 20 µL. Quantitative polymerase chain reaction (qPCR) was performed using 5×HOT FIREPol^®^ EvaGreen qPCR Mix Plus (Solis BioDyne, Tartu, Estonia). The PCR amplification mix contained 2 µL of cDNA template, 1 µL of primers (0.5 µL each at 10 pmol/mL), 3 µL of PCR Master Mix, and 9 µL of dd H_2_O. The reaction conditions were as follows: initial denaturation at 95 °C for 15 min, denaturation at 95 °C for 15 s, annealing at 60 °C for 20 s, and elongation at 72 °C for 20 s (40 cycles) [27]. Specific primers for the expression analysis of *BDNF* and *TrkB* genes, as well as the endogenous control genes glyceraldehyde-3-phosphate dehydrogenase (*GAPDH*) and peptidylprolyl isomerase C (*PPIC*), were designed using Primer3 software (The Whitehead Institute, Boston, MA, USA) (Table 1). Amplification specificity was further validated via electrophoresis of the obtained amplicons on a 2% agarose gel and visualized under a UV light camera. Data were analyzed using Rotor Gene 6000 v. 1.7 software (Qiagen, Hilden, Germany) containing a comparative quantification option and Relative Expression Software Tool, based on the PCR efficiency correction algorithm developed by Pfaffl et al. [32,33]. Gene expression levels were normalized to the geometric mean of the expression of reference genes. Endogenous control genes were assayed in each sample to compensate for variation in the cDNA concentration and PCR efficiency between individual tubes [27].

### 2.6. Statistical Analysis

Initially, all data were assessed using the Shapiro–Wilk normality test and subsequently grouped into parametric and non-parametric groups. Tissue BDNF concentrations were analyzed using one-way analysis of variance (STATISTICA, Stat Soft, Tulsa, OK, USA), followed by the post-hoc Last Significance Difference test. Statistical evaluations of differences in mRNA expression levels for BDNF and its TrkB receptor in the examined brain structures between treatment groups were performed using non-parametric statistics, involving the Kruskal–Wallis test with multiple comparisons of average ranks and the Mann–Whitney *U* test for pairwise group comparisons. Differences were considered significant at *p* < 0.05, and all data are presented as the mean ± standard error of the mean (SEM).

## 3. Results

### 3.1. Tissue BDNF Concentration

**Figure 1:**
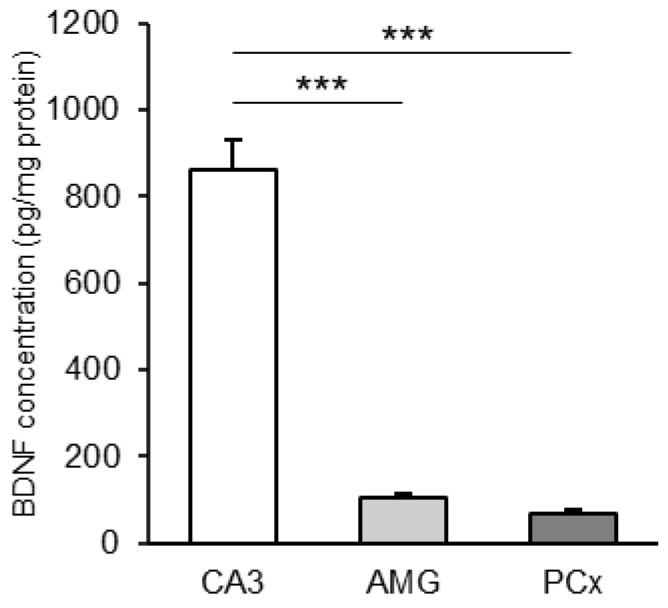
Brain-derived neurotrophic factor (BDNF) protein concentration (pg/mg protein, mean ± SEM) in homogenates of the examined sheep brain structures: CA3 field of the hippocampus (CA3), amygdala (AMG), and the prefrontal cortex (PCx). Significance of differences: ***, *p* < 0.001.

Due to the larger volume of collected tissue material, the BDNF concentration was examined in the CA3, AMG, and PCx. Of these tissues, the highest concentration of the peptide was found in the hippocampal CA3 field (*p* < 0.001). The BDNF concentration in the CA3 was 8-fold higher than the concentration recorded in the AMG and 12-fold higher than in the PCx (Figure 1).

**Figure 2:**
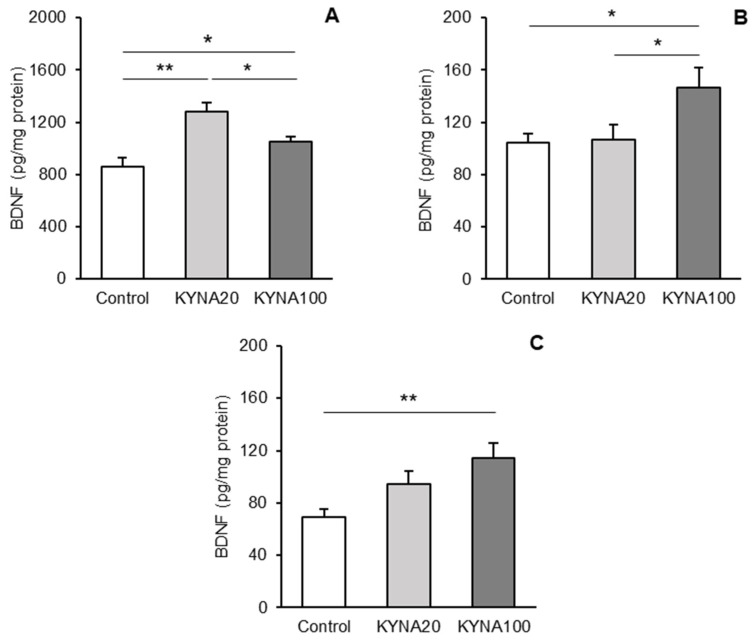
Comparison of brain-derived neurotrophic factor (BDNF) protein concentration (pg/mg protein, mean ± SEM) in homogenates of the hippocampal CA3 field (**A**), amygdala (**B**), and the prefrontal cortex (**C**) in sheep infused with control solution and the lower (total 20 μg, (KYNA20) and higher (total 100 μg, KYNA100) doses of kynurenic acid (KYNA) into the third brain ventricle. Significance of differences: *, *p* < 0.05; **, *p* < 0.01.

Differences in BDNF levels between treatment groups are depicted for individual tissues in Figure 2A–C. Both doses of KYNA caused a significant increase in the BDNF concentration in the hippocampal CA3 field (*p* < 0.01 and *p* < 0.05 for KYNA20 and KYNA100, respectively), compared to the control group (Figure 2A). The increase was more pronounced after the infusion of KYNA20 compared to KYNA100 (*p* < 0.05). In contrast, KYNA20 had no significant effect on BDNF levels in the AMG and PCx, whereas KYNA100 significantly increased the BDNF concentration in both tissues (AMG—*p* < 0.05, and PCx—*p* < 0.01) compared to controls (Figure 2B and 2C, respectively).

### 3.2. Relative Abundance of BDNF and TrkB mRNA

*BDNF* and *TrkB* gene transcripts were detected in the hippocampal CA3 field, AMG, and PCx, as well as in the selected hypothalamic areas, the MBH, and the POA. Due to the standardization of BDNF and TrkB mRNA contents relative to the control group, performed separately using individual tissues, differences in transcript levels between tissues were assessed visually. The highest BDNF mRNA expression was observed in the MBH, followed by the PCx, POA, AMG, and CA3. The highest abundance of TrkB mRNA was observed in the AMG, followed by the MBH, PCx, CA3, and POA.

**Figure 3:**
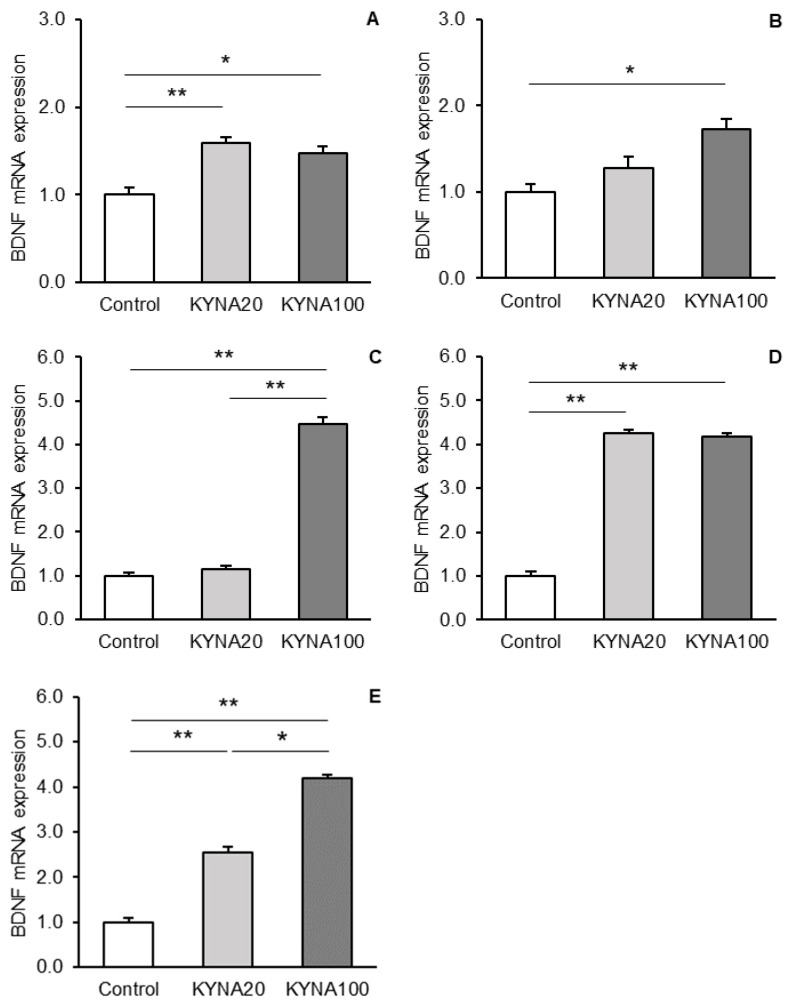
Comparison of relative brain-derived neurotrophic factor (BDNF) mRNA expression (mean ± SEM) in the hippocampal CA3 field (**A**), amygdala (**B**), prefrontal cortex (**C**), in the hypothalamic medial-basal area (**D**), and the preoptic area (**E**) in sheep infused with control solution and the lower (total 20 μg, (KYNA20) and higher (total 100 μg, KYNA100) doses of kynurenic acid (KYNA) into the third brain ventricle. Significance of differences: *, *p* < 0.05; **, *p* < 0.01.

The differences in the abundances of *BDNF* gene transcripts between the treatment groups are shown for individual tissues in Figure 3A–E. Both doses of KYNA caused a significant increase in the abundance of BDNF mRNA in the hippocampal CA3 field with *p* < 0.01 for KYNA20 and *p* < 0.05 for KYNA100 compared to the control group (Figure 3A). However, there was no significant difference in BDNF mRNA levels between the KYNA20 and KYNA100 groups. In the AMG (Figure 3B) and PCx (Figure 3C), only the KYNA100 dose significantly increased the BDNF transcript abundance (*p* < 0.05 and *p* < 0.01, respectively) compared to controls. With respect to the MBH (Figure 3D), both KYNA doses had a comparable stimulatory effect (*p* < 0.01) on the expression of the *BDNF* transcript. However, the effect was more gradual in the POA (Figure 3E); while both doses significantly increased BDNF mRNA expression (*p* < 0.01) compared to controls, the KYNA100 group showed a higher (*p* < 0.05) *BDNF* transcript abundance than the KYNA20 group.

**Figure 4:**
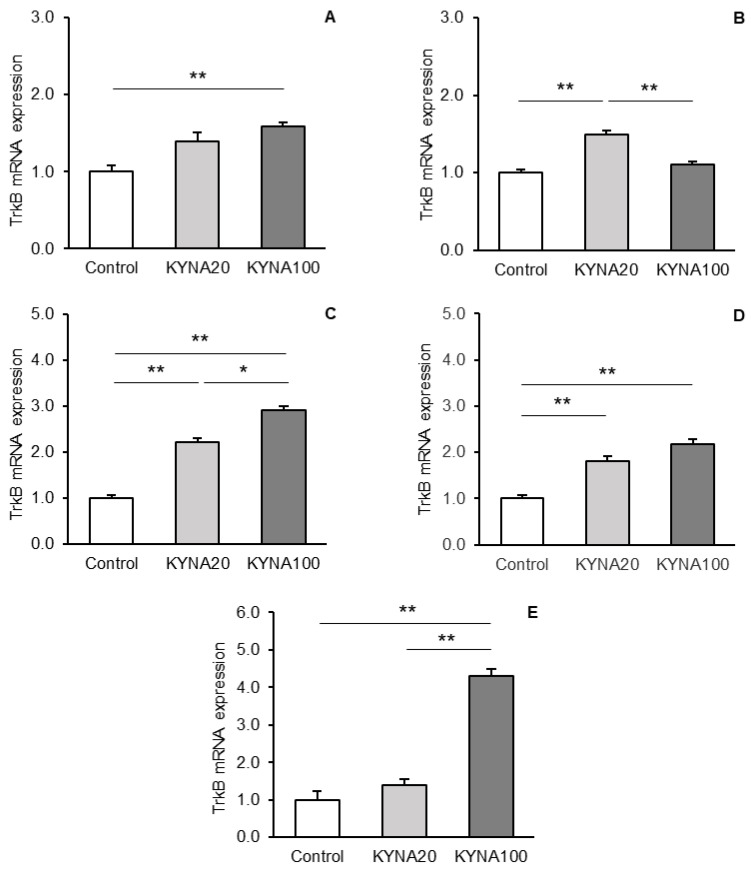
Comparison of relative tropomyosin-related kinase receptor B (TrkB) mRNA expression (mean ± SEM) in the hippocampal CA3 field (**A**), amygdala (**B**), prefrontal cortex (**C**), in the hypothalamic medial-basal area (**D**), and the preoptic area (**E**) in sheep infused with control solution and the lower (total 20 μg, (KYNA20) and higher (total 100 μg, KYNA100) doses of kynurenic acid (KYNA) into the third brain ventricle. Significance of differences: *, *p* < 0.05; **, *p* < 0.01.

The differences in the abundances of *TrkB* gene transcripts between the treatment groups are shown for individual tissues in Figure 4A–E. Both doses of KYNA induced an increase in the *TrkB* transcript levels in the hippocampal CA3 field (Figure 4A); however, only the KYNA100-infused sheep showed a statistically significant increase (*p* < 0.01) compared to controls. With respect to the AMG (Figure 4B), the KYNA20 dose proved most effective, significantly increasing (*p* < 0.01) TrkB mRNA levels. A differential response to KYNA infusion was observed in PCx tissue (Figure 4C), as both doses significantly (*p* < 0.01) increased TrkB mRNA expression compared to the control, but the transcript abundance was higher (*p* < 0.05) in the group of sheep treated with KYNA100 than those administered KYNA20. Intracerebroventricular KYNA infusion also exerted a stimulatory effect on TrkB mRNA expression in various areas of the hypothalamus. Both KYNA doses caused a significant (*p* < 0.01) increase in *TrkB* transcript abundance in the MBH (Figure 4D) compared to controls. In the POA (Figure 4E), however, a significant increase (*p* < 0.01) in transcript levels occurred only in response to the KYNA100 dose.

## 4. Discussion

Many therapeutic agents used to treat various CNS disorders and diseases have been shown to affect BDNF signaling. It appears that beneficial changes in neurotrophin expression can also be induced by modulating the levels and activity of endogenously derived compounds within the CNS. The present work revealed a positive association between increasing KYNA levels in the CSF and the expression of BDNF and its specific receptor TrkB in various brain structures responsible for vital, cognitive, and psychological functions. Most of these structures are located in close proximity to the ventricular system and, due to the multidirectional CSF circulation [22,34], may be easily exposed to infused KYNA. Although doses of administered KYNA appear to exceed physiological concentrations described for the mammalian brain [25,35], it is worth noting that a significant portion of KYNA may flow out of the third ventricle with the mainstream and be absorbed into capillaries, e.g., in the median eminence, other circumventricular organs, or the choroid plexus. Additionally, 30 min intervals between infusions facilitated the removal of the administered substance from the infusion site, preventing the accumulation of its high concentrations. According to Stone et al. [36], the concentration of KYNA at the target site is particularly important. While at the point of release, KYNA concentrations can reach millimolar levels or higher, they decrease with distance due to the dilution in physiological fluids. Research has demonstrated that similar concentrations of KYNA (approximately 1 mM) administered to the cerebroventricular circulation in sheep can trigger various receptor-activated responses in the targeted tissues [23,31].

One key finding of our study is the high concentration of BDNF protein in the CA3 field of the sheep hippocampus, despite the relatively lower abundance of BDNF mRNA in the same region. This observation is consistent with a previous result in rats, where both complementary DNA (cDNA) labeling and BDNF-immunoreactivity were shown to be relatively dense in nearly all cells of this hippocampal region [37]. Rodent studies have further demonstrated that both mossy fibers (MFs), originating from the DG presynaptically, and CA3 pyramidal cells postsynaptically contain elevated levels of BDNF and also express TrkB [37,38]. According to Griego and Galvan [39], BDNF–TrkB signaling plays a fundamental role as a homeostatic regulator controlling the intrinsic excitability of the CA3 network. The observed discrepancy between BDNF mRNA and protein levels likely reflects the different locations of synthesis sites in the nerve cells and the presence of anterograde axonal transport [37,38]. The relatively lower levels of available mRNA transcripts for BDNF and its receptor in relation to other structures may indicate the high tissue requirement for BDNF protein and its involvement in signaling processes. Nevertheless, the high concentrations of BDNF in the CA3 homogenate found in our study reflect high quantities of mature peptide both stored and released locally.

In mammals, the hippocampal CA3 region plays a specific role in memory processes and spatial orientation and is highly susceptible to neurodegeneration. The pyramidal neurons, which largely define the morphology of the CA3 field, form extensive dendritic trees characterized by high plasticity. This internal connectivity enables the processing of input signals from the cortex (via the perforant path) and DG (via MF), as well as excitatory transmissions to a large number of neurons in the CA1 field (via Shaffer collaterals) and other adjacent hippocampal areas [40]. Ji and coworkers [41] demonstrated that BDNF could induce neurite elongation or branching through distinct signaling cascades, depending on the concentration of BDNF in the extracellular space. Moreover, BDNF was shown to promote neurite differentiation to axons in vivo and was required for axon formation in cultured hippocampal neurons [42,43]. Extensive evidence indicates that activity-induced increases in long-term synaptic potentiation (LTP), a cellular correlate of learning and memory, are strongly linked to efficient BDNF–TrkB signaling [44]. Studies in TrkB- or BDNF-deficient mice have shown that impaired BDNF–TrkB signaling leads to the significant downregulation of LTP in the hippocampus; on the other hand, such an insufficiency can be reversed by restoring *BDNF* gene expression or by administering recombinant BDNF [44]. While the detailed mechanisms by which BDNF modulates LTP in the hippocampus are beyond the scope of this work, it is noteworthy that BDNF is also expressed in hippocampal astrocytes. This glial expression may contribute to enhancing neuronal firing efficiency to some extent [45]. It should be mentioned that the postnatal generation of new neurons (adult neurogenesis) observed in the DG is another morphological correlate of neuronal hippocampal plasticity [6]. In the SGZ, newborn cells migrate a short distance to the inner layer of granule cells, where they differentiate into granule neurons. Subsequently, they extend long axonal projections along the MF pathway and reach the target CA3 pyramidal cell layer. Various growth and trophic factors, including BDNF, significantly contribute to the proliferation, survival, and development of newborn neurons in the adult hippocampus and other neurogenic regions [46].

Both the hippocampus and AMG are integral components of the limbic system, connected through an extensive and reciprocal network, reaching, e.g., the hypothalamus and frontal cortex. In sheep, as in other mammals, the AMG consists of groups of nuclei clustered mainly in three subregions: centromedial, basolateral, and cortical, which have been implicated in a wide variety of functions, such as emotions, motivation, learning, or memory [47]. Although several neurotrophins and their cognate receptors have been identified in the amygdaloid nuclei, BDNF–TrkB signaling in the AMG seems to be particularly important for fear learning [48]. The examined AMG sections represent all three subregions, which showed moderate levels of both BDNF protein and BDNF mRNA, along with relatively high expression of mRNA for the TrkB receptor. Studies in rodents indicate that BDNF–TrkB signaling predominates in synaptic sites of neurons entering the AMG from thalamic and cortical regions [37,38]. Reciprocal connections of the limbic system and thalamus also extend to the PCx, which plays a key role in cognitive control and executive functions, including decision-making and stress control ability [49]. In humans, the PCx shares extensive connections with the AMG, which are crucial for processing emotional stimuli, particularly negative ones, and are influenced by varying BDNF levels [50]. Thus, although the PCx is remote from the cerebroventricular system, the activation of BDNF–TrkB signaling in this structure in response to KYNA infusion may result from its strong interconnections with the limbic system. Research on the specific function of the PCx in sheep remains, however, limited. An early work, using stressors, such as isolation or repeated transportation, mainly showed the effects of stress on the emotional reactivity of young animals [51]. Another study demonstrated that the prenatal aversive handling of ewes resulted in significant changes in apical dendritic spine density and morphology in the hippocampus and PCx of one-month-old offspring [52]. In rodent studies, chronic stress-induced damage to synaptic plasticity, including dendritic atrophy, synapse reduction, and volumetric changes in the PCx, has been associated with reduced BDNF expression [53]. Dysfunction in the limbic system and PCx, as well as deficits in cognitive control due to an insufficient supply of neurotrophic factors, are believed to be a key cause of many mental and neurological disorders in humans. Accordingly, reduced BDNF levels have been reported in the brains of patients with depression [54] and schizophrenia [55]. In the context of our research, it is important to note that prenatal redirection of kynurenine metabolism to KYNA has been shown to enhance neuronal excitability and LTP and increase the expression of several neurodevelopmental proteins in the brain of rat offspring [56]. Another study on rodents showed an antidepressant-like effect of 7-chlorokynurenic acid, associated with the induction of BDNF–TrkB signaling in the limbic system in mice subjected to chronic unpredictable mild stress [57]. Additionally, in an elderly human population with mood disorders, a tryptophan-rich diet increased both tryptophan and KYNA urinary levels, exerting a beneficial, antidepressant effect on mental health and improving the metabolism of this amino acid [58]. On the other hand, enhanced cognitive abilities and synaptic plasticity, associated with increased extracellular glutamate levels, were observed in mice with a targeted deletion of kynurenine aminotransferase II, a key enzyme involved in KYNA biosynthesis in the brain [59]. Interesting outcomes also emerged after the exposure of experimental animals to KYNA during adolescence, which led to increased sensitivity to reward-related cues and impaired LTP later in life [60]. The data presented indicate that the kynurenine pathway plays a fundamental role in the early development of the CNS. Variations in KYNA concentrations may, depending on age and the presence of harmful factors, modulate the ability of brain structures to process signals by engaging neurotrophic factors.

Important BDNF-dependent neuronal centers, such as the arcuate, dorsomedial, and ventromedial (VMH) nuclei, are located within the MBH, which, along with the lateral hypothalamic area, play critical roles in regulating food intake and body weight [37,61]. BDNF has been shown to act as an anorexigenic factor, influencing these processes through its interaction with a variety of locally produced signaling proteins [59]. Moreover, Ameroso et al. [62] identified VMH astrocytes as essential cellular substrates for BDNF in terms of maintaining energy and glucose homeostasis. The POA, on the other hand, with its complex organization, contains sites critical for regulating body temperature, the electrolyte balance, and the wake–sleep cycle [63]. In addition, the medial POA is one of the most important areas for controlling instinctive behaviors, including parental care, mating, and aggression [64]. In many mammals, including sheep, both the MBH and POA are also regions housing gonadotropin-releasing hormone (GnRH) neurons, which trigger the synthesis and release of pituitary gonadotropins [63]. The involvement of BDNF in the regulation of central reproductive functions was previously demonstrated in sheep by Przybył et al. [65], who observed substantial changes in kisspeptin and GnRH mRNA expression after the intracerebroventricular administration of BDNF. However, the specific role of KYNA as a primary regulator of the described BDNF activity in the hypothalamus has not been fully explored. Research indicates that disturbances in the kynurenine pathway associated with certain neuropsychiatric disorders may be related to an individual’s nutritional status, e.g., as seen in cases of anorexia [66]. Conversely, obesity may impair the synthesis of glutamate NMDA receptor subunits, which could be targets of both substances [67]. Equally important for our research is the fact that the hypothalamus in adults emerged as a new neurogenic region with substantial proliferative capacity, constitutively generating cells of the neuronal lineage [68]. Although the level of neurogenesis in the hypothalamus is lower than that observed in well-established hippocampal neurogenic regions, its significance lies in the critical functional implications of this brain region. In general, BDNF expression in different areas of the hypothalamus is associated with maintaining the control of neuroendocrine functions and plays a vital role in numerous aspects of hypothalamic control over key physiological processes [61,62,65,69].

Studies have demonstrated that BDNF transcription and release are mainly stimulated by excitatory synaptic activity, especially involving ionotropic glutamate NMDA receptors [70]. Antagonistic compounds can induce fast antidepressant-like effects, associated with the disinhibition of glutamate transmission, leading to a transient increase in glutamate levels and, consequently, enhanced BDNF expression [71]. Moreover, the involvement of AMPA receptors has also been shown to be involved in the upregulation of BDNF expression in the CNS [72]. Since many brain regions receive glutamatergic inputs, the involvement of KYNA, as a modulator of glutamate receptors and related physiological processes, especially at micromolar concentrations, seems reasonable. Therefore, maintaining appropriate KYNA levels in the brain, depending on the age and disease state, could be considered part of the therapy for some CNS disorders and conditions. However, a critical aspect that requires comprehensive research is the permeability of the blood–brain barrier/blood–cerebrospinal fluid barrier (BBB/BCSFB) to KYNA. According to some researchers, the efficiency of KYNA in penetrating these barriers is low, indicating that its concentration in the CNS relies on local synthesis [22]. On the other hand, evidence exists that the peripheral administration of KYNA can produce effective central effects. Scharfman and Goodman [73] demonstrated that the hippocampal responses following peripheral KYNA administration were qualitatively similar to those observed with direct administration to hippocampal slices. According to Heyes and Quearry [74], the slight increase in KYNA concentrations in the CSF, following systemic KYNA administration, could result from the heightened sensitivity of the BBB/BSCFB to the increased availability of KYNA in the blood. Such specific cases require further investigations.

## 5. Conclusions

The present study demonstrated a positive association between KYNA levels in the cerebroventricular circulation and BDNF–TrkB expression in various brain regions in a sheep model. This finding suggests that a transient increase in the CSF KYNA concentration can potentially restore BDNF synthesis, of which deficiency underlies numerous neurological disorders.

## Figures and Tables

**Table 1:** Specific primers used for gene expression analysis.

GENE	PRIMERS (5′–3′)	GENBANK ACC. NO.	AMPLICON SIZE
*BDNF*	F: CGTTGGCTGACACTTTTGAA R: CGCAGCATCCAGGTAATTTT	XM_012143442.1	188
*TRKB*	F: TGTCTGAGCTGATCCTGGTG R: TATCTGCAGGTTTGCCAGTG	XM_012117231.2	155
*GAPDH*	F: GGGTCATCATCTCTGCACCT R: GGTCATAAGTCCCTCCACGA	NM_001190390.1	131
*PPIC*	F: TGGAAAAGTCGTGCCCAAGA R: TGCTTATACCACCAGTGCCA	XM_004008676.1	158

BDNF: brain-derived neurotrophic factor, TrkB: tyrosine kinase receptor B, GAPDH: glyceraldehyde-3-phosphate dehydrogenase, PPIC: peptidylprolyl isomerase C, F: forward primer, R: reverse primer. Real-time PCR amplification efficiency of target and reference genes was 96–100%.

## Data Availability

The datasets analyzed during the current study are available from the corresponding author upon reasonable request.
